# A cleaner burning biomass-fuelled cookstove intervention to prevent pneumonia in children under 5 years old in rural Malawi (the Cooking and Pneumonia Study): a cluster randomised controlled trial

**DOI:** 10.1016/S0140-6736(16)32507-7

**Published:** 2016-12-07

**Authors:** Kevin Mortimer, Chifundo B Ndamala, Andrew W Naunje, Jullita Malava, Cynthia Katundu, William Weston, Deborah Havens, Daniel Pope, Nigel G Bruce, Moffat Nyirenda, Duolao Wang, Amelia Crampin, Jonathan Grigg, John Balmes, Stephen B Gordon

**Affiliations:** Malawi Liverpool Wellcome Trust Programme, Blantyre, Malawi; Liverpool School of Tropical Medicine, Liverpool, UK; Malawi Liverpool Wellcome Trust Programme, Blantyre, Malawi; Malawi Liverpool Wellcome Trust Programme, Blantyre, Malawi; Malawi Epidemiology and Intervention Research Unit, Chilumba, Malawi; Malawi Epidemiology and Intervention Research Unit, Chilumba, Malawi; Malawi Liverpool Wellcome Trust Programme, Blantyre, Malawi; Liverpool School of Tropical Medicine, Liverpool, UK; Liverpool School of Tropical Medicine, Liverpool, UK; University of Liverpool, Liverpool, UK; University of Liverpool, Liverpool, UK; Malawi Epidemiology and Intervention Research Unit, Chilumba, Malawi; London School of Hygiene & Tropical Medicine, London, UK; Liverpool School of Tropical Medicine, Liverpool, UK; Malawi Epidemiology and Intervention Research Unit, Chilumba, Malawi; London School of Hygiene & Tropical Medicine, London, UK; Queen Mary University of London, London, UK; University of California, Berkeley, CA, USA; University of California, San Francisco, CA, USA; Malawi Liverpool Wellcome Trust Programme, Blantyre, Malawi; Liverpool School of Tropical Medicine, Liverpool, UK

## Abstract

**Background:**

WHO estimates exposure to air pollution from cooking with solid fuels is associated with over 4 million premature deaths worldwide every year including half a million children under the age of 5 years from pneumonia. We hypothesised that replacing open fires with cleaner burning biomass-fuelled cookstoves would reduce pneumonia incidence in young children.

**Methods:**

We did a community-level open cluster randomised controlled trial to compare the effects of a cleaner burning biomass-fuelled cookstove intervention to continuation of open fire cooking on pneumonia in children living in two rural districts, Chikhwawa and Karonga, of Malawi. Clusters were randomly allocated to intervention and control groups using a computer-generated randomisation schedule with stratification by site, distance from health centre, and size of cluster. Within clusters, households with a child under the age of 4·5 years were eligible. Intervention households received two biomass-fuelled cookstoves and a solar panel. The primary outcome was WHO Integrated Management of Childhood Illness (IMCI)-defined pneumonia episodes in children under 5 years of age. Efficacy and safety analyses were by intention to treat. The trial is registered with ISRCTN, number ISRCTN59448623.

**Findings:**

We enrolled 10 750 children from 8626 households across 150 clusters between Dec 9, 2013, and Feb 28, 2016. 10 543 children from 8470 households contributed 15 991 child-years of follow-up data to the intention-to-treat analysis. The IMCI pneumonia incidence rate in the intervention group was 15·76 (95% CI 14·89–16·63) per 100 child-years and in the control group 15·58 (95% CI 14·72–16·45) per 100 child-years, with an intervention versus control incidence rate ratio (IRR) of 1·01 (95% CI 0·91–1·13; p=0·80). Cooking-related serious adverse events (burns) were seen in 19 children; nine in the intervention and ten (one death) in the control group (IRR 0·91 [95% CI 0·37–2·23]; p=0·83).

**Interpretation:**

We found no evidence that an intervention comprising cleaner burning biomass-fuelled cookstoves reduced the risk of pneumonia in young children in rural Malawi. Effective strategies to reduce the adverse health effects of household air pollution are needed.

**Funding:**

Medical Research Council, UK Department for International Development, and Wellcome Trust.

## Introduction

Almost half of the world's population, including 700 million Africans, rely on biomass fuels for cooking (eg, animal dung, crop residues, wood, and charcoal).^1^–^3^ Although a billion people in sub-Saharan Africa are projected to gain access to electricity by 2040, 530 million will remain dependent on biomass fuels.^4^ Biomass fuel is typically burned in open fires, often indoors, leading to high levels of household air pollution from smoke. Women and children experience high exposures in and around the home due to gender-based domestic roles and these exposures have been linked to a range of adverse health outcomes, including chronic obstructive pulmonary disease, lung cancer, ischaemic heart disease, asthma, and pneumonia.^1^–^3^ Household air pollution from biomass fuel smoke is a leading cause of global disability and mortality with over 4 million deaths attributed to this exposure annually by WHO.^3^,^5^ This burden, including about half a million deaths due to pneumonia in young children, falls disproportionately on the poor, especially in sub-Saharan Africa. In Malawi, pneumonia is a leading cause of mortality among children younger than 5 years of age.^6^,^7^ Over 95% of households depend on biomass for fuel and household air pollution levels are high and well beyond WHO safe limits.^8^,^9^ A substantial burden of pneumonia in young children might be attributable to biomass smoke exposure in this setting.

Two trials to assess the effects of interventions to reduce exposure to biomass smoke on health outcomes have been published to date.^10^,^11^ Both trials used stoves that reduce exposure mainly by venting emissions to the outdoor environment with a chimney rather than by improving combustion efficiency. Romieu and colleagues^10^ compared a Patsari stove intervention with the traditional open fire on respiratory symptoms and lung function in 552 women in Mexico. Adherence to the intervention was poor but the Patsari stove reduced respiratory symptoms (eg, rate ratio [RR] 0·29 [95% CI 0·11–0·77] for wheeze) and lung function decline (31 mL *vs* 62 mL over 1 year; p=0·01) in those who used the stove. The RESPIRE trial randomised 534 households with a pregnant woman or infant in highland Guatemala to a Plancha stove (with good compliance) or open fire and assessed the impact on pneumonia in children younger than 19 months.^11^ In intention-to-treat analyses the Plancha gave a non-significant reduction in incidence of physician-diagnosed pneumonia (primary outcome) (RR 0·84 [95% CI 0·63–1·13]; p=0·26; after multiple imputation to adjust for higher referral compliance among intervention homes, RR 0·78 [0·59–1·06]; p=0·10) and a significant reduction in physician-diagnosed severe pneumonia (RR 0·67 [0·45–0·98]; p=0·04). There was a 50% reduction in personal child exposures (measured by carbon monoxide) but this improvement still left exposure levels well above WHO recommended limits.^11^ Cleaner burning biomass-fuelled cookstoves are now available that improve combustion efficiency by incorporating technologies (eg, electric fans and gasifier re-burning of evolved gases) that lead to a substantial, up to 90%, reduction in emissions in laboratory testing.^12^

Access to cleaner cooking fuels and technologies is limited by many factors in the developing world although poverty is a key underlying issue for most.^13^ The Global Alliance for Clean Cookstoves (GACC) was launched in 2010 to tackle the lack of access to clean affordable energy through public–private partnerships. A central aim of GACC is for 100 million homes to adopt clean cookstoves and fuels by 2020.^14^ However, there is limited evidence to assess the potential health benefits of such an approach: globally there are no data from clinical trials of cleaner burning biomass-fuelled interventions and in Africa there are no data from clinical trials of any cookstove interventions.^3^ We set out to determine whether a cleaner burning biomass-fuelled cookstove intervention would reduce pneumonia incidence in children under 5 years of age living in rural Malawi compared with continuation of traditional open fire cooking (see video).

## Methods

### Study design and participants

We did a cluster randomised controlled trial with two groups of equal size comparing the effects of a cleaner burning biomass-fuelled cookstove intervention with continued use of open fire cooking on pneumonia incidence in children under 5 years of age living in rural Malawi over a 2 year period (the Cooking and Pneumonia Study, CAPS). We defined 150 community-level clusters within villages across two districts of Malawi where we had established field research sites: Chikhwawa in the southern Shire river valley and Karonga on the northern Malawi lakeshore. The Malawi College of Medicine Research Ethics Committee (ethics committee reference number P.11/12/1308) and the Liverpool School of Tropical Medicine Research Ethics Committee (ethics committee reference number 12.40) approved the protocol, which was peer reviewed, and a summary was published by *The Lancet*.^15^

Following community engagement exercises with village leaders and communities and the identification of a representative for each cluster, households with at least one child aged up to 4·5 years were invited to participate. Written informed consent (or witnessed thumbprint for those unable to read and write) was obtained at cluster and household level (parent or guardian of child) before participation. The trial was open to all consenting households with a usually resident child in the eligible age range. Households that became eligible for inclusion during the course of the trial (through birth, adoption, or in-migration) were recruited until 6 months before the trial ended.

### Randomisation and masking

Clusters were randomly allocated to intervention and control groups using a computer-generated randomisation schedule with stratification by site, distance from health centre, and size of cluster. Randomisation was done by the trial statistician using dummy codes A and B to represent intervention and control groups; to ensure the statistician remained masked, the identity (allocation) of A and B was determined by a second, independent statistician. The trial statistician used a dummy randomisation code to develop statistical programs for data analyses before unblinding the trial data.

### Procedures

Intervention households received two cleaner burning biomass-fuelled cookstoves (Philips HD4012LS; Philips South Africa, Johannesburg, South Africa), a solar panel to charge the battery for the stove fan, and user training. A fan incorporated into these cookstoves improves combustion efficiency; smoke emissions have been found to be reduced by around 90% compared with the open fire in laboratory testing.^12^ Cookstoves were repaired and replaced as needed. Stove use monitors were placed on one of the stoves in a randomly selected 10% sample of intervention households at baseline and at the 12 month visit to provide an objective measure of use, by recording temperature fluctuations. Control households continued using traditional cooking methods (typically open fires). At the start of the trial control households were informed they would receive the intervention at the end of the study period for equity and to maximise retention. Each household was visited every 3 months by fieldworkers although by the time the 21 month visit was due we were 3 months behind schedule and as such moved directly on to the final 24 month visit. Community engagement events were done regularly across all clusters to maintain engagement, facilitate discussion, and troubleshoot.

### Outcomes

The primary outcome was the incidence of WHO Integrated Management of Childhood Illness (IMCI)-defined pneumonia in children under 5 years of age diagnosed by physicians, medical officers, or other appropriately trained staff at local health-care facilities routinely accessed by trial participants, unaware of intervention allocation.^16^

Secondary outcomes were the incidence of all pneumonia including those not meeting IMCI criteria, severe pneumonia, and death in children under 5 years of age. Additional outcomes included: the association between exposure to household air pollution and the development of pneumonia in children under 5 years of age in rural Malawi; prevalence and determinants of obstructive lung disease in adults in rural Malawi and the relationship between exposure to household air pollution and the rate of decline in lung function in adults in rural Malawi; affordability and cost-effectiveness of the intervention from household, health-care system, and societal perspectives; and what can be learned from trial participants and non-participants about adoption of the intervention that could inform effective implementation of the trial findings in the future. The findings of these additional outcomes will be reported separately.

We provided training and equipment including respiratory rate timers, thermometers, and pulse oximeters to support local health services in delivering high quality clinical assessments and documentation of pneumonia diagnoses and severity assessments.^17^ A supply of antibiotics was provided when local health service stocks ran out.

The IMCI pneumonia assessment protocol was used because chest radiographs were not consistently available and were not part of the usual diagnostic work-up in rural Malawi. Non-severe IMCI pneumonia was defined as the presence of cough or difficulty breathing and fast breathing (60, 50, or 40 breaths per min or higher in those aged <2 months, 2–12 months, and 1–5 years, respectively). Severe IMCI pneumonia was defined additionally by chest in-drawing, stridor, or any general danger sign (inability to drink or breastfeed, vomiting, convulsions, lethargy, or unconsciousness). Oxygen saturation of less than 90% was included as an additional and objective marker of severity.

In Malawi there is a patient-held medical record system. Patients present their Malawi Ministry of Health and Population health passport whenever they seek medical care. All trial participants were provided with a new health passport if they did not already have one with sufficient blank pages. A sticker was inserted explaining that the child was in the trial; a brief summary of the IMCI pneumonia assessment protocol; and boxes to tick about clinical criteria and severity if the patient was diagnosed with pneumonia. Malaria was tested with a rapid diagnostic test and treated as indicated, as part of routine clinical practice. During or after the attendance, the trial team was notified about diagnoses of pneumonia and deaths by text or phone call from health facility based staff or a member of the household using a phone and airtime credit provided to each CAPS cluster representative.

Fieldworkers reviewed and captured digital images of the health passports of all children in the trial at visits every 3 months to obtain information about episodes of pneumonia and deaths not otherwise reported. Electronic case report forms programmed using Open Data Kit (ODK; Department of Computer Science and Engineering, University of Washington, Seattle, WA, USA) software and administered by smartphone (Samsung Galaxy S3) were used to collect these data. Data were transferred from smartphones to a secure server at each study site, checked, uploaded to a database, cleaned, and then relayed onwards to a central secure server in preparation for analysis. Source document verification was done for every case of pneumonia using digital images of health records as source documents by a single member of the study team, the methodology for which was reviewed and approved at a Pan African Thoracic Society meeting in Malawi in July, 2016.^18^

Data about cooking-related adverse events were collected at field visits every 3 months. Adverse events that met seriousness criteria (death, life-threatening, led to hospitalisation or prolongation of an existing hospitalisation, or led to disability or incapacity) were reported immediately to the trial coordinating centre for assessment of causality, seriousness, and expectedness, and for appropriate medical action and onward reporting to be arranged. An independent data monitoring committee reviewed all potentially cooking-related serious adverse events.

### Statistical analysis

We aimed to include 150 clusters representing an estimated 10 600 eligible children. Clusters were randomised in equal numbers to two intervention groups to provide, over the whole study period, about 21 200 child-years of follow-up and 90·3% power to detect a reduction in the rate of pneumonia from five per 100 child-years in the control group to four per 100 child-years in the intervention group (proportionally, a 20% reduction). This conservative estimate of incidence was used to allow for potential reductions in the rate of pneumonia following pneumococcal vaccine introduction. We assumed an effective intra-cluster correlation (ICC) of 0·10 and a coefficient of variation in cluster size of 30–35%. Full sample size considerations are available online.

All primary analyses used intention-to-treat principles; the intention-to-treat population was all participants with at least one follow-up record before reaching 5 years of age. Secondary per-protocol analyses were also done; the per-protocol population included all participants 54 months of age or younger at recruitment and, in the intervention group, participants from households with at least one follow-up record at which stove use was reported. Generalised estimating equation (GEE) modelling methods were used to assess the primary outcome, adjusting for clustering effects. Covariate adjusted analysis was done on the primary outcome by adding the following prespecified variables into the above GEE model: district, age of child at recruitment, sex, distance to nearest health centre, number of children younger than 5 years living in household, number of people in household who smoke regularly, other sources of fire or smoke (other than cooking) to which child was exposed on a daily (or almost daily) basis, socioeconomic status of household, number of previous pneumonia episodes experienced by child, and status of childhood vaccinations. Secondary outcomes were analysed similarly using GEE models; time to event data were analysed using Cox regression model with frailty for the cluster effect. One masked interim safety analysis was done and reviewed by the data monitoring committee to determine whether there were grounds to stop the trial for safety. Provision for an interim analysis for efficacy was included in the protocol although was not required by the data monitoring committee. SAS statistical package (version 9.3) was used. The full peer-reviewed statistical analysis plan is available online. The study is registered with ISRCTN, number ISRCTN59448623.

### Role of the funding source

The funders had no role in the study design, data collection, analysis, interpretation, or writing of the report. The corresponding author had full access to all the study data and had final responsibility for the decision to submit for publication.

## Results

We enrolled 10 750 children from 8626 households across 150 community-level clusters in two sites in rural Malawi between Dec 9, 2013, and Feb 28, 2016. 207 children were lost to follow-up (103 from the intervention group and 104 from the control group) from 156 households (83 in the intervention group and 73 in the control group) between baseline and first follow-up visit (figure). Data were therefore available from 10 543 children (5297 in the intervention group and 5246 in the control group) from 8470 households (4256 in the intervention group and 4214 in the control group) for at least one follow-up visit and form the intention-to-treat and safety dataset (figure). Baseline characteristics were balanced between intervention and control groups (table 1). There were 15 991 child-years of follow-up (7964 child-years from the intervention group and 8027 child-years from the control group). The last participant follow-up visit was on Sept 14, 2016.

We excluded 47 children (21 in the intervention group and 26 in the control group) because they were aged over 54 months at recruitment and 25 children from intervention households due to self-reported never use of the intervention to create the per-protocol population. The per-protocol population was therefore 10 471 children (5251 in the intervention group and 5220 in the control group) from 8429 households (4224 in the intervention group and 4205 in the control group).

There were 2506 episodes of IMCI pneumonia in the overall intention-to-treat population (1255 in the intervention group and 1251 in the control group) giving an incidence rate of 15·67 (95% CI 15·06–16·28) per 100 child-years. The incidence rate in the intervention group was 15·76 (95% CI 14·89–16·63) per 100 child-years and in the control group 15·58 (95% CI 14·72–16·45) per 100 child-years, with an incidence rate ratio (IRR) of 1·01 (95% CI 0·91–1·13; p=0·80). The estimated ICC was 0·03. After adjustment for baseline values, the IRR for IMCI pneumonia for the intervention versus control was 1·05 (95% CI 0·93–1·18; p=0·44). The incidence rate for IMCI pneumonia in the overall per-protocol population was 15·70 (95% CI 15·09–16·32) per 100 child-years with an intervention versus control IRR of 1·02 (95% CI 0·91–1·14; p=0·77). The hazard ratio for time from enrolment to first pneumonia episode in the intervention versus control group was 1·06 (95% CI 0·93–1·21; p=0·29). The incidence rate for all pneumonia episodes including those not meeting IMCI criteria was 17·21 (95% CI 16·57–17·85) per 100 child-years with an intervention versus control IRR of 1·02 (95% CI 0·91–1·13; p=0·75).

In the intervention versus control group the IRR for severe pneumonia episodes defined by IMCI criteria was 1·30 (0·99–1·71; p=0·06), for oxygen saturation less than 90% 1·56 (0·70–3·46; p=0·28), and for death 0·76 (0·17–3·37; p=0·71; table 2). A rapid diagnostic test for malaria was done at the time of IMCI pneumonia diagnosis in 1547 cases, of which 473 (30·6%) were positive.

At the first (3 months), 12, and 24 month follow-up visits, 73·3% (2535 of 3457), 59·0% (1889 of 3204), and 49·8% (1414 of 2841) of intervention households, respectively, reported that the cookstoves met all their cooking needs. Most households that answered an optional follow-up question reported using the stoves for at least one meal a day on average over the 2 years of follow up (table 3). The most common reason given for not using the cookstoves for all cooking tasks was that the cookstoves had malfunctioned; battery failures were particularly common. We placed 364 stove use monitors on one of the two cookstoves in households selected for this sub-study from which 537 (36·9%) of a potential 1456 sets of usable data were obtained during year 1 and 498 (45·6%) of a potential 1092 sets for year 2. The mean number of cooking events per day for the monitored cookstove was 0·51 (SD 0·55) during year 1 and 0·34 (0·40) during year 2. Over the course of the trial, repairs or replacements were done on 13 192 (3·1 per intervention household) occasions for cookstoves and 5259 (1·2 per intervention household) occasions for solar panels. Control households were provided with two advanced cookstoves (ACE-1; African Clean Energy Company, Maseru, Lesotho) with a solar charger and solar lamp (part of the standard cookstove package by this time) at the end of the trial.

596 serious adverse events were reported excluding pneumonia. With the exception of a borderline statistically significant increased risk of malaria in the intervention group (IRR 1·32 [95% CI 1·03–1·69]; p=0·03) there were no differences in incidence rates of serious adverse events between the intervention and control groups (table 4). Of these, 19 (nine in the intervention group and ten in the control group) were cooking-related burns (IRR 0·91 [95% CI 0·37–2·23]; p=0·83). Additionally, 1505 burns (549 in the intervention group and 956 in the control group) not meeting seriousness criteria were reported (IRR 0·58 [95% CI 0·51–0·65]; p<0·0001). There were 53 deaths (malaria [14], pneumonia [seven], sepsis [five], malnutrition [five], accidental injury [four], gastroenteritis [one], burns [one], and other or unknown [16]) from the time of enrolment, 44 (25 in the intervention group and 19 in the control group) of which occurred in the intention-to-treat population (IRR 1·32 [95% CI 0·73–2·40]; p=0·36). The data monitoring committee reviewed trial safety data in March, 2015, and found no grounds to stop the trial early.

## Discussion

This community-level cluster randomised controlled trial in Malawi is the largest trial of a cookstove intervention on health outcomes to date. Malawi is one of the world's poorest countries and within particularly impoverished rural areas it is frequent for households to have insufficient money at times to buy basic items like soap or food for their normal meals. The intention-to-treat analysis included data from 10 543 children from 8470 households across 150 community-level clusters representing 15 991 child-years of follow-up data. The number of children included was close to our original sample size estimate of 10 600 although the total follow-up time was less than the estimated 21 200 child-years. The incidence rate of IMCI pneumonia was high (15·67 per 100 child-years) and about three times higher than the deliberately conservative assumption used for our sample size calculation (five per 100 child-years). The observed incidence rate was more in agreement with 2010 estimates of IMCI pneumonia incidence in Malawi (24·3 per 100 child-years).^19^ Baseline characteristics were well balanced between the intervention and control groups. The intention-to-treat and per-protocol analyses with and without adjustment for baseline data were consistent in finding no effect of the intervention on the incidence of IMCI pneumonia in the young children in the study. Although limited by a smaller number of cases, analyses restricted to severe pneumonia episodes similarly found no beneficial effect of the intervention.

The intervention comprised a package of two cleaner burning biomass-fuelled cookstoves to provide two cooking hobs, a solar charger for the battery in the cookstove, a repair and replacement service, and user training. The intervention cookstove was selected on the basis of being among the cleanest burning biomass-fuelled cookstoves available at the time we designed the trial, with 90% lower emissions than open fires in laboratory tests.^12^ The repair and replacement service was heavily used, with each intervention household requiring this service four times on average over the course of the trial. The high rate of cookstove and solar charger malfunction was surprising since these products had been specifically designed and developed for the indications, end users, and environments in which we assessed them. Failure of the rechargeable lead acid batteries in the cookstoves was a particularly common problem requiring replacement of the batteries. This issue could have been largely avoided had the cookstoves had better quality batteries. Cookstove malfunction was the most common reason given by households for not using the cookstoves for all cooking needs. Although we did repairs and replacements as quickly as possible there were inevitably short periods when households only had one functioning cookstove. When this alone was not sufficient for all cooking needs the household would have had to revert to using the open fire. Nevertheless, and despite these challenges, self-reported use of the cookstoves was probably as high as could be realistically expected with three-quarters of intervention households reporting the use of the stoves for all their cooking needs at 3 months, reducing to about half of households after 2 years; as is true in kitchens around the world there is no single cooking device suitable for every task.

We included stove use monitors on one of two cookstoves in a subset of intervention households to provide an objective measure of use. Although these monitors generated less usable data than anticipated due to technical problems, and their presence on just one of the two stoves might have affected which stove was used, they similarly indicate a waning in use over time. The impact on pneumonia in children might have been greater had adoption of the intervention been higher but the levels of adoption we achieved are probably higher than would be the case outside the context of a trial, particularly if an intensive and free repair and replacement service was not available.

Baseline data showed that in addition to almost exclusive use of biomass fuels for cooking, exposure to smoke from other sources including burning of rubbish, tobacco, and income generation activities was a common day-to-day experience. Additionally, as cookstoves were only issued to households that had a resident child younger than 5 years, exposure to smoke from neighbours’ or relatives’ cooking fires is likely. It is thus possible that these other air pollution exposures would have negated any potential beneficial impact of the cookstove intervention and that had even cleaner burning cookstoves been used, they would be unlikely to have had an impact. Personal exposure and exposure-response data, to be published separately, will provide additional insights here.

There was a high incidence of serious adverse events unrelated to cooking across the trial population reflecting the expected range and frequency of serious childhood illnesses seen in rural Malawi. There was a borderline statistically significant increased risk of malaria in the intervention versus the control group, which might be a false positive finding reflecting multiplicity of secondary safety analyses. Data from elsewhere suggest smoke from open fire cooking is unlikely to protect against malaria vectors.^20^ Nevertheless, this is an area of uncertainty and this observation warrants further investigation. Serious adverse events related to cooking (all burns) resulted in considerable morbidity and one death in our study population. Less serious burns were a common occurrence with 508 (4·8%) of 10 543 children reporting a burn in the 3 months before study enrolment and 1505 burns not meeting seriousness criteria reported during the study. Although serious burns were evenly distributed across the trial groups, there was a substantial 42% reduction (IRR 0·58 [95% CI 0·51–0·65]; p<0·0001) in risk of non-serious burns in the intervention group, suggesting the intervention offers considerable safety advantages over the open fire.

The findings of our trial add substantially to the trials of chimney stoves in Mexico (552 women followed up for 10 months) and Guatemala (518 children from 534 households contributing 560 child-years of follow-up).^10^,^11^ Although none found a statistically significant effect on the primary health outcome of interest, all point to benefits of the interventions in secondary analyses—there was a reduction in burns in Malawi, a reduction in respiratory symptoms, and decline in lung function in adults in a per-protocol analysis of intervention adopters in Mexico, and a reduction in severe acute lower respiratory tract infections in Guatemala.

Although the design of our trial was necessarily open, considerable effort was put into minimising the risk of bias associated with a non-blinded study. Specifically, we provided training to fieldworkers to ensure participants were treated in the same way irrespective of trial group; scheduled the same frequency offield visits to intervention and control villages; collected primary outcome data in the same way for intervention and control villages; provided training to local health-care workers about how to make an IMCI diagnosis of pneumonia, the importance of carrying out assessments, and treatment based on clinical need and without enquiring about trial allocation to achieve blinded assessment of the primary outcome; and did blinded independent source document verification for all pneumonia diagnoses. The trial was done in a challenging environment where poverty is widespread and health services are stretched. We used the widely adopted WHO IMCI diagnostic criteria for pneumonia implemented by independent health-care workers because this approach is most reflective of the reality on the ground where access to more sophisticated diagnostic tools such as chest radiographs are not consistently available. Although this pragmatic approach might lack specificity, it is the best measure of the entity reported as pneumonia and associated with morbidity and mortality among children under 5 years of age in Malawi and elsewhere in sub-Saharan Africa.

In conclusion, we found no evidence of a reduction in pneumonia in young Malawian children in a rigorously conducted clinical trial of a cleaner burning biomass-fuelled cookstove intervention. The lack of effect on pneumonia might be explained by exposure to other sources of air pollution, including rubbish burning and tobacco smoke, that could have overwhelmed any potential effects of the cookstoves. It is also possible that the cookstoves used simply did not reduce emissions sufficiently to have an effect. An important implication of these observations is that tackling any individual source of air pollution exposure in isolation is unlikely to be effective for improving health; an integrated approach to achieving clean air that tackles rubbish disposal, tobacco smoking, and other exposures, as well as robust cleaner cooking solutions (eg, cleaner stoves and fuels) that achieve a high rate of acceptance is probably needed to deliver health benefits.^21^

## Supplementary Material

Supplementary video

## Figures and Tables

**Figure:**
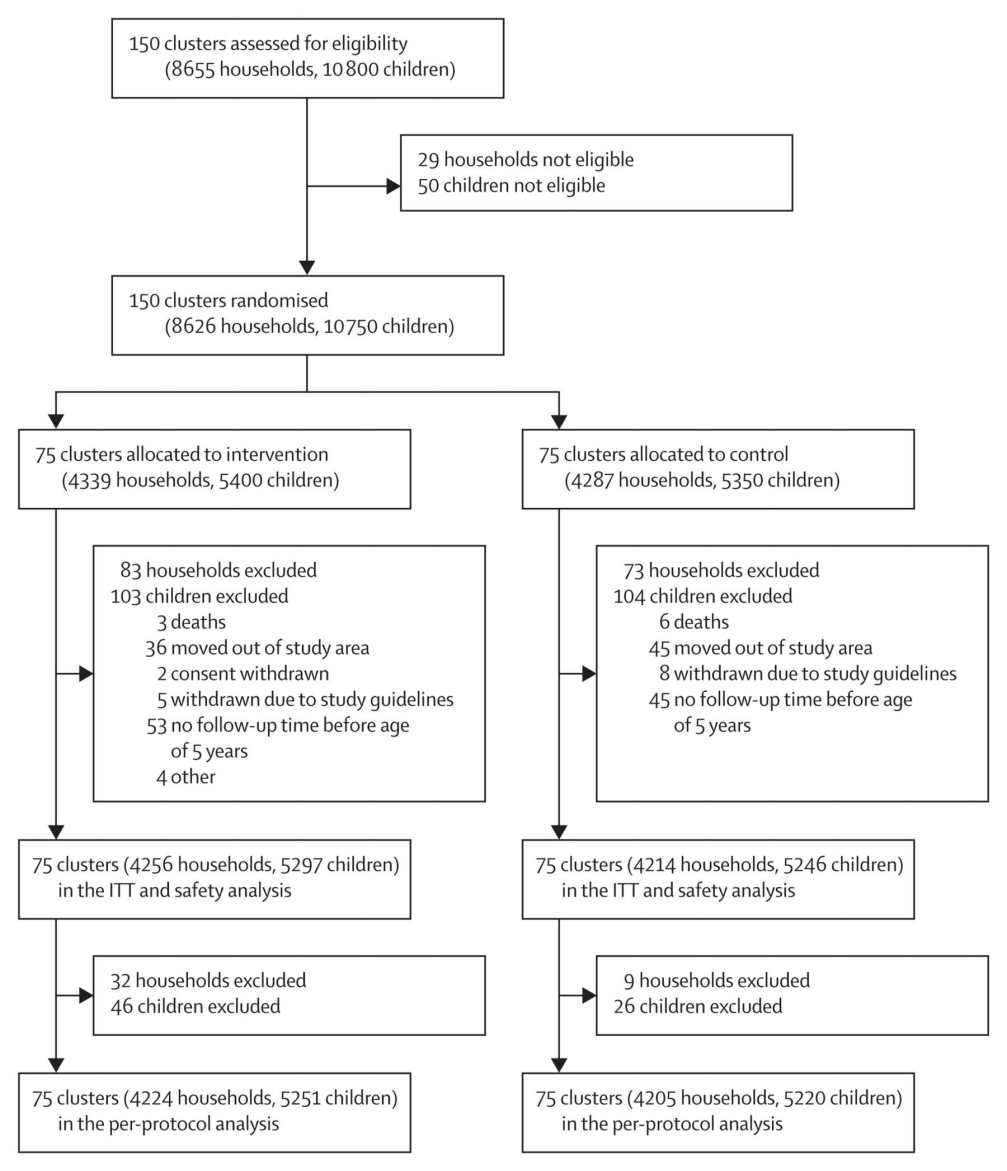
Trial profile ITT=intention to treat.

**Table 1:** Baseline characteristics of the intention-to-treat population

	Intervention	Control
**Individual-level data**		
Number of children	5297	5246
Age (months)	24·5 (16·1)	23·8 (16·1)
Sex		
Male	2641 (49·9%)	2561 (48·8%)
Female	2656 (50·1%)	2685 (51·2%)
Vaccination status (course completed)*		
Diphtheria, tetanus, pertussis, hepatitis B and *Haemophilus influenzae* B	2893 (79·4%)	2980 (79·4%)
Pneumococcal conjugate	2122 (58·3%)	2239 (59·7%)
Polio	2726 (74·8%)	2857 (76·1%)
Rotavirus	1713 (47·0%)	1774 (47·3%)
Measles	2166 (59·5%)	2228 (59·4%)
Had pneumonia at least once in the preceding 12 months†	762 (15·4%)	844 (17·4%)
Had a cooking-related burn in the preceding 3 months	219 (4·1%)	289 (5·5%)
**Household-level data**		
Number of households	4256	4214
Fuel used regularly for cooking‡		
Wood	2494 (58·6%)	2390 (56·7%)
Crop residues	1339 (31·5%)	1320 (31·3%)
Charcoal	563 (13·2%)	723 (17·2%)
Dung	20 (0·5%)	25 (0·6%)
Electricity	5 (0·1%)	13 (0·3%)
Paraffin/kerosene	2 (0·0%)	6 (0·1%)
Gas	0	2 (0·0%)
Other	2 (0·0%)	0
Tobacco smoker in the household	746 (17·5%)	634 (15·0%)
Daily or almost daily exposure to smoke from:‡		
Burning rubbish	1747 (41·0%)	1677 (39·8%)
Cooking for business	554 (13·0%)	513 (12·2%)
Brick production	177 (4·2%)	214 (5·1%)
Paraffin/kerosene lamps	87 (2·0%)	105 (2·5%)
Mosquito coils	44 (1·0%)	82 (1·9%)
Beer production	54 (1·3%)	44 (1·0%)
Other sources	63 (1·5%)	47 (1·1%)
Source of drinking water‡		
Bore hole	1900 (44·6%)	1973 (46·8%)
Tap to house	401 (9·4%)	378 (9·0%)
Shared communal tap	389 (9·1%)	324 (7·7%)
Covered well	254 (6·0%)	233 (5·5%)
Lake or river	255 (6·0%)	157 (3·7%)
Open well	191 (4·5%)	253 (6·0%)
Other	3 (0·1%)	1 (0·0%)
Toilet facilities		
Simple pit latrine	3448 (81·0%)	3553 (84·3%)
None	763 (17·9%)	612 (14·5%)
Water toilet	37 (0·9%)	23 (0·5%)
Ventilated improved pit	8 (0·2%)	26 (0·6%)
Experienced a time in the last year when there was not enough food for the household to have its normal meals	2131 (50·1%)	2113 (50·1%)
Experienced a time in the last year when the household did not have money to buy bathing soap	2608 (61·3%)	2597 (61·6%)

Data are mean (SD) or n (%).

* Vaccination status available for 7395 children (3642 in the intervention group and 3753 in the control group).

† Data available for 9798 children (4954 in the intervention group and 4844 in the control group).

‡ Household could give multiple responses.

**Table 2:** Incidence rate and incidence rate ratio of severe pneumonia defined according to IMCI criteria, oxygen saturation less than 90%, or death

	Intervention (n=5297)		Control (n=5246)		Incidence rate ratio (95% CI)	p value
	Number of cases	Incidence rate (cases per 100 child-years, 95% CI)	Number of cases	Incidence rate (cases per 100 child-years, 95% CI)		
IMCI criteria	186	2·33 (2·00–2·67)	145	1·80 (1·51–2·09)	1·30 (0·99–1·71)	0·06
Oxygen saturation <90%	17	0·21 (0·11–0·31)	11	0·14 (0·06–0·22)	1·56 (0·70–3·46)	0·28
Death	3	0·04 (0·00–0·08)	4	0·05 (0·00–0·10)	0·76 (0·17–3·37)	0·71

IMCI=WHO Integrated Management of Childhood Illness.

**Table 3:** Meals cooked using intervention cookstoves at 3, 12, and 24 month follow-up visits

	3 months (n=922)	12 months (n=1315)	24 months (n=1427)
None	73 (7·9%)	333 (25·3%)	312 (21·9%)
1 meal per day	248 (26·9%)	339 (25·8%)	524 (36·7%)
1–2 meals per day	213 (23·1%)	301 (22·9%)	215 (15·1%)
2 or more meals per day	388 (42·1%)	342 (26·0%)	376 (26·3%)

**Table 4:** Incidence rate and incidence rate ratio of serious adverse events excluding pneumonia

	Intervention (n=5297)		Control (n=5246)		Incidence rate ratio (95% CI)	p value
	
	Number of events	Incidence rate (cases per 100 child-years, 95% CI)	Number of events	Incidence rate (cases per 100 child-years, 95% CI)
Malaria	175	2·19 (1·87–2·52)	134	1·66 (1·38–1·95)	1·32 (1·03–1·69)	0·03
Sepsis	51	0·64 (0·46–0·82)	54	0·67 (0·49–0·85)	0·95 (0·64–1·41)	0·81
Gastroenteritis	24	0·30 (0·18–0·42)	28	0·35 (0·22–0·48)	0·86 (0·49–1·53)	0·62
Burns	9	0·11 (0·04–0·19)	10	0·12 (0·05–0·20)	0·91 (0·37–2·23)	0·83
Accidental injury	12	0·15 (0·07–0·24)	9	0·11 (0·04–0·18)	1·42 (0·58–3·45)	0·44
Malnutrition	5	0·06 (0·01–0·12)	5	0·06 (0·01–0·12)	1·01 (0·29–3·48)	0·99
Asthma	6	0·08 (0·02–0·14)	2	0·02 (0·01–0·06)	3·03 (0·51–18·11)	0·22
Other	36	0·45 (0·30–0·60)	36	0·45 (0·30–0·59)	1·02 (0·62–1·66)	0·94
Total	318	3·99 (3·55–4·43)	278	3·45 (3·05–3·86)	1·15 (0·95–1·38)	0·15

## Research in context

### Evidence before this study

Results of two trials assessing the effects of interventions to reduce exposure to biomass smoke on health outcomes were published before this study. Both tested stoves that reduce exposure mainly by venting emissions to the outdoor environment via a chimney rather than by improving combustion efficiency. Romieu and colleagues compared a chimney stove intervention with traditional open fire on respiratory symptoms and lung function in 552 women in Mexico. Adherence to the intervention was poor but the chimney stove reduced respiratory symptoms and lung function decline in those who used the stove. The RESPIRE trial randomised 534 households with a pregnant woman or infant in highland Guatemala to a chimney stove (with good compliance) or open fire and assessed the impact on pneumonia in children younger than 19 months. In intention-to-treat analyses, there was a non-significant reduction in incidence of physician-diagnosed pneumonia (primary outcome) in the chimney stove group (rate ratio [RR] 0·84 [95% CI 0·63–1·13]; p=0·26) and a significant reduction in physician-diagnosed severe pneumonia (RR 0·67 [0·45–0·98]; p=0·04).

### Added value of this study

This trial is the largest study of the effects of a biomass-fuelled cookstove intervention on health outcomes to date. The trial was done in very resource-poor populations in rural Malawi where pneumonia is one of the leading causes of morbidity and mortality in young children and almost all households burn biomass fuels for cooking. Data from 10 543 children from 8470 households across 150 community-level clusters contributed 15 991 child-years of follow-up data to the intention-to-treat analysis. The intervention comprising two biomass-fuelled cookstoves, a solar charger, repair and maintenance service, and user training had no effect on the risk of pneumonia in children under the age of 5 years in rural Malawi.

### Implications of all the available evidence

There is insufficient evidence on which to recommend replacing open fires with biomass-fuelled cookstoves for the prevention of pneumonia in young children in settings where biomass fuels are burned in open fires for cooking.
